# Predictors of functional impairment and awareness in people with dementia, mild cognitive impairment and healthy older adults from a middle-income country

**DOI:** 10.3389/fpsyt.2022.941808

**Published:** 2022-07-28

**Authors:** Larissa Hartle, Daniel C. Mograbi, Helenice Charchat Fichman, Camila Assis Faria, Maria Angélica Sanchez, Pricila C. C. Ribeiro, Roberto Alves Lourenço

**Affiliations:** ^1^Department of Psychology, Pontifícia Universidade Católica, Rio de Janeiro, Brazil; ^2^Department of Psychology, Institute of Psychiatry, Psychology and Neuroscience, King’s College London, London, United Kingdom; ^3^Department of Internal Medicine, Faculty of Medical Sciences, Rio de Janeiro State University, Rio de Janeiro, Brazil; ^4^Department of Psychology, Faculty of Philosophy and Humanities, Federal University of Minas Gerais, Belo Horizonte, Brazil; ^5^Department of Medicine, Pontifícia Universidade Católica, Rio de Janeiro, Brazil

**Keywords:** dementia, MCI, ADL, cognition, awareness, dependence, functional impairment

## Abstract

**Objective:**

To investigate the demographic, clinical and cognitive correlates of functional capacity and its awareness in people with dementia (PwD; *n* = 104), mild cognitive impairment (PwMCI; *n* = 45) and controls (healthy older adults; *n* = 94) in a sample from a middle-income country.

**Methods:**

Dementia and MCI were diagnosed, respectively, with DSM-IV and Petersen criteria. Performance in activities of daily living (ADL) at three different levels [basic (The Katz Index of Independence), instrumental (Lawton instrumental ADL scale) and advanced (Reuben’s advanced ADL scale)], measured through self- and informant-report, as well as awareness (discrepancy between self- and informant-report), were compared between groups. Stepwise regression models explored predictors of ADL and their awareness.

**Results:**

PwD showed impairment in all ADL levels, particularly when measured through informant-report. No differences were seen between controls and PwMCI regardless of measurement type. PwD differed in awareness of instrumental and basic, but not of advanced ADL, compared to controls. Age, gender, education and fluency were the most consistent predictors for ADL. Diagnosis was a significant predictor only for instrumental ADL. Awareness of basic ADL was predicted by memory, and awareness of instrumental ADL was predicted by general cognitive status, educational level, and diagnosis.

**Conclusion:**

Results reinforce the presence of lack of awareness of ADL in PwD. Use of informant-reports and cognitive testing for fluency are suggested for the clinical assessment of ADL performance. Finally, assessment of instrumental ADL may be crucial for diagnostic purposes.

## Introduction

Functional capacity, i.e., the ability to perform activities of daily living (ADL), is an important variable in the context of aging, being affected by a variety of chronic conditions linked to older age. Loss of functional ability is commonly associated with cognitive decline in older adults (1), and can be the distinguishing diagnostic criterion between dementia and mild cognitive impairment (2) (MCI), both being characterized by cognitive decline. It is known that there is a progressive loss of functional capacity in the course of dementia, with deficits in cognition and other abilities affecting the capacity to perform daily activities (3–5). Nevertheless, previous studies provided conflicting results in relation to the extent to which functional capacity is affected at each moment of the condition, probably due to the heterogeneity of patients under the same diagnosis but on different stages of the illness and with different degrees of cognitive impairment (6–9).

One important issue when considering the relationship between ADL performance and cognitive impairment is the existence of different levels of functional capacity according to the complexity of the activity (10). Typically, three levels are suggested: basic ADL, including simple self-care duties such as bathing and eating; instrumental activities, involving more complex tasks such as handling money and preparing meals; and advanced activities, including social life and hobbies. Advanced activities are the first to be impaired with the appearance of cognitive impairment (11, 12), but functional decline progresses until it affects even basic activities (1, 3). Any level of impairment can cause disability and, without the development of compensatory strategies to offset these difficulties, lead to dependence and decreases in the quality of life of people affected and their caregivers (13).

The notion of a hierarchy of ADL is particularly important for the diagnosis of MCI. Initially, it was believed that all levels of ADL would be preserved in this condition (2). However, studies have found that it may be possible to see subtle changes (12, 14), especially in more complex advanced activities (3, 11). It is possible that some measures are not sensitive enough to capture these subtle changes, especially when the activity can still be completed even when the subject makes mistakes (15, 16). Few studies have explored these small variations, but the ones that do suggest that performance accuracy will decrease with the escalation of cognitive impairment until the ability to live independently becomes fully compromised (15).

Studies that analyze the cognitive processes related to loss of functional capacity vary in their conclusions. In general, some evidence suggests that executive functioning is the best predictor of functional capacity (3, 8), but there is also evidence that deterioration in the ability to perform everyday tasks could be related to a general cognitive impairment (17). It is also useful to differentiate between commission errors (performing a step incorrectly—using salt instead of sugar to make a cake) and omission errors (not performing a step—not using sugar at all), with only the latter error being related to a deficit in general cognitive resources (17). Other studies found that omission errors seem to be also linked to memory impairment (14, 15). Generally, studies that search for cognitive correlates of functional capacity focus on one function, one diagnosis or one ADL level at a time, instead of combining all of them. Advanced ADL are the least studied (3).

One potential issue leading to heterogeneity of results may be that cognitively impaired patients do not fully acknowledge the extent to which they have functional impairments. This lack of awareness about the diagnosis and its consequences has been termed anosognosia. Although findings are mixed, it has been shown that people with MCI (PwMCI) may have limited awareness about their abilities (18). A recent meta-analysis, for example, suggested that there already is mild anosognosia of cognitive abilities in PwMCI, and that it becomes more severe in dementia (19), despite the relationship between awareness and dementia severity not being linear (20, 21). Lack of awareness was mainly measured by comparing informant- and self-reports in identified studies (19) and results suggest that the use of informant-based measures assessing functional abilities may be relevant not only to PwD, but also to PwMCI.

A better understanding of the association between cognitive impairment, ADL performance and its awareness is critical to aid the development of better interventions, rehabilitation programs and compensatory strategies. These studies are especially relevant for low- and middle-income countries (LMIC), considering the scarcity of data from these world regions in comparison to developed countries (22). Studies in LMIC may also shed light on the specific contribution of sociodemographic variables to ADL. For example, lower educational level, and consequently poorer executive functions performance, may impact functional capacity.

Accordingly, the current study aimed to investigate functional capacity and awareness in dementia and MCI with a sample from a LMIC. Specifically, three levels of ADL were explored (advanced, instrumental and basic), measured both by self- and informant-report. In addition to exploring differences between patient groups, sociodemographic and cognitive correlates for each type of ADL and awareness of functional ability were also investigated.

## Materials and methods

### Participants and setting

**FIGURE 1:**
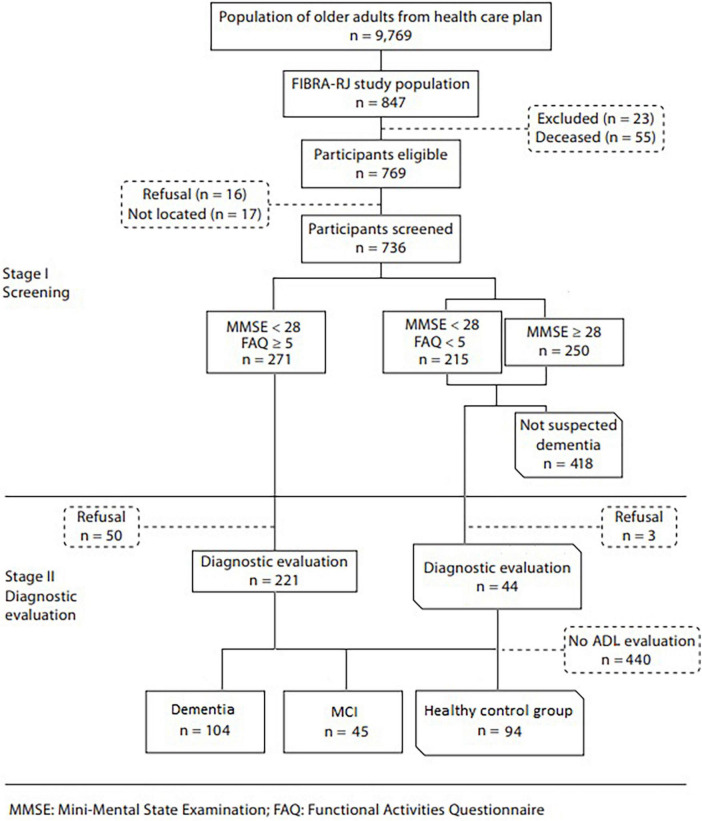
Flowchart of the FIBRA study.

The sample for this study was obtained from the Frailty in Brazilian Older People—Rio de Janeiro Section (FIBRA Study) stage II database (23, 24). FIBRA study was organized in two stages: screening for cognitive impairment (I) and diagnostic evaluation (II). A flowchart can be seen in Figure 1. During stage I, a gender and age-stratified sample from a Brazilian private healthcare plan received home visits by trained research assistants and had their cognitive performance and functional capacity assessed, respectively, by the Mini-Mental State Examination (25, 26) (MMSE) and the Functional Activities Questionnaire (27, 28) (FAQ). The criteria for inclusion in the FIBRA study were having been a client of the health care plan for at least 12 months, being at least 65 years old, and a resident in one of the districts of the North Zone of Rio de Janeiro City.

During stage I, the sample was divided into two groups based on MMSE and FAQ performances. The first one—250 subjects with MMSE ≥ 28, and 215 with MMSE < 28, but without loss of functional capacity (FAQ < 5)—were regarded as cognitively unimpaired and thus negative for dementia syndrome and MCI. From this group, a sample (*n* = 44) was drawn randomly for evaluation in Stage II, to check for the presence of false negatives (none of them were diagnosed as having dementia). The second group—271 subjects with MMSE scores < 28 and FAQ ≥ 5—were considered suspected of having dementia or MCI and were invited to be assessed by clinical and neuropsychological evaluations (stage II). The assessment was carried out by a multidisciplinary team led by a geriatrician.

The eligibility criteria defined for inclusion of participants in Stage II—diagnostic evaluation, in addition to their MMSE score as described above, were their score on the FAQ. They were contacted by telephone and referred for a comprehensive geriatric assessment, which included cognitive and functional evaluation. The diagnosis of dementia syndrome and MCI was established by consensus among geriatricians and neuropsychologists according to the Diagnostic and Statistical Manual of Mental Disorder-Fourth Edition (29) and Petersen (30) criteria, respectively. The diagnosis also relied on laboratory tests and neuroimaging and neuropsychological tests (instruments section below) (23).

In order to take part in the current study, all participants needed to have an informant living or being in close contact with them for at least 10 years and being at least 23 years old [such that informants would have been at least adolescents (13 + years) when their contact initiated]. Some participants had missing informant or self-report measures of ADL and were also excluded.

The present study analyzed data from 243 participants who fulfilled all these criteria. Based on these assessments, the sample was split into the following subgroups: 104 participants diagnosed with dementia; 45 diagnosed with MCI and 94 healthy older adults (hereafter, control participants). Although information about dementia subtype was not fully available for the dementia group, most had a diagnosis of Alzheimer’s Disease.

### Instruments

#### Cognitive abilities

General cognitive level was measured through the MMSE (25, 26), with scores ranging from 0 to 30. Episodic memory was assessed through the Rey Auditory Verbal Learning Test (31), focusing on immediate (A1) and delayed recall (A7). Working memory was measured with the digit span test from the Wechsler Adult Intelligence Scale (32), with total scores calculated as the sum of the direct and reverse digit span. Finally, fluency was assessed through the semantic (total number of animals named) and phonemic fluency (total number of words named with the letters FAS) tasks (33, 34). As an informant measure of cognitive decline, the IQCODE (35, 36) was used. The questionnaire contains 26 items and seeks to retrospectively verify change in an elderly everyday cognitive function.

#### Activities of daily living

Activities of daily living (ADL) were assessed through three different instruments. For all instruments, self-report and informant report were obtained. Informants were people in close contact with the participants (see previous section), typically relatives (partners and children).

##### Advanced activities of daily living

Were measured with Reuben’s advanced ADL scale (37). This 12-item questionnaire assesses independent functioning and participation in activities such as traveling and taking part in cultural events, clubs, political events and religious institutions. Activities that were lost or never tried were scored as 0, and preserved activities were scored as 1. Total scores ranged from 0 to 10.

##### Instrumental activities of daily living

Were measured with the Lawton instrumental ADL scale (38, 39). The scale assesses the ability to use the phone, shop, travel alone, prepare meals, do housework and handle finances and medication. Total scores ranged from 8 to 21, with higher scores indicating more preserved abilities.

##### Basic activities of daily living

Were measured with The Katz Index of Independence in ADL. The scale has six items assessing bathing, dressing, personal hygiene, feeding, mobility and continence (40, 41). To keep consistency with the other ADL measurements, the scale was reverse scored, such that higher scores indicate more preserved abilities. Total scores ranged from 6 to 18.

#### Awareness of functional ability

Awareness was assessed through the discrepancy between self-report and informant-report, a method widely used in the literature (42). For each ADL questionnaire (advanced, instrumental and basic), informant-report was subtracted from self-reported, such that positive scores indicate overestimation of ability.

### Statistical analysis

Data analysis was carried out using SPSS software (version 20.0). Descriptive statistics were used to illustrate the sample characteristics, with differences between groups being tested with one-way ANOVAs, followed by *post hoc t*-tests, or a chi-square test in the case of gender.

For each ADL type (advanced, instrumental and basic), differences between groups (PwD, MCI and controls) were calculated with one-way ANOVAs, followed by pairwise comparisons adjusted with Bonferroni corrections. This was done for self-report, informant-report and discrepancy scores (awareness variables).

Finally, stepwise regression models were calculated to explore predictors of functional capacity and awareness. Predictors included demographic (educational level, gender and age), clinical (diagnostic group) and cognitive variables (MMSE score, digit span, phonemic fluency, categorical fluency, RAVLT immediate memory and delayed recall). Models were run separately for each type of ADL (advanced, instrumental or basic) and variable [self-, informant-report, discrepancy score (awareness)]. In all models, to avoid inflation of type II error and exclusion of predictors involved in suppressor effects, we used a backward regression method. The best models were selected on the basis of a trade-off between highest explained variance (*R*^2^), highest cross-validity (adjusted *R*^2^) and Akaike’s Information Criterion (AIC).

## Results

### Sample characteristics

The demographic characteristics and clinical profile of the sample are described in Table 1. There were significant differences in age between groups [*F*(2, 240) = 27.94, *p* < 0.001], with PwD being older than controls and MCI group (*p* < 0.001). Educational level was also significantly different between groups, [*F*(2, 239) = 8.72, *p* < 0.001], with fewer years of education in the PwD group in relation to controls. There were no differences in terms of gender distribution between groups [χ^2^(2) = 3.14, *p* = 0.214].

**TABLE 1:** Sociodemographic and clinical characteristics of participants.

Variable	Dementia (*n* = 104) Mean (*SD*), range	MCI (*n* = 45) Mean (*SD*), range	Control group (*n* = 94) Mean (*SD*), range
Age (mv = 0)	85.1 (7.4), 67–101	79.2 (6.9), 67–92	77.9 (7.5), 67–98
Education (mv = 2)	7.1 (5.3), 0–20	8.7 (4.8), 0–18	10.2 (5.2), 0–22
Gender*(mv = 0)	81/23	30/15	64/30
MMSE (mv = 12)	18.0 (6.5), 0–28	25.6 (3.5), 14–30	27.2 (2.4), 18–30
IQCODE (mv = 65)	4.0 (0.6), 3.0–5.0	3.4 (0.2), 3.0–4.0	3.2 (0.2), 2.8–4.0
RAVLT			
Immediate recall (mv = 49) Delayed recall (mv = 51)	23.2 (7.6), 10–45 2.1 (2.3), 0–10	32.0 (9.1), 9–49 4.7 (2.5) 0–9	38.9 (9.5), 15–60 6.8 (3.0), 0–14
Digit Span (mv = 45)	8.9 (2.9), 2–16	10.7 (3.3), 3–17	12.4 (3.3), 5–23
Digit to symbol (mv = 71)	11.8 (9.0), 0–35	27.4 (13.4), 5–72	28.7 (14.6), 1–69
CAMCOG (mv = 61)	57.7 (15.9), 18–93	78.7 (11.0), 42–100	82.9 (10.4), 44–101
Verbal fluency			
Categorical (mv = 48) Phonemic (mv = 49)	18.8 (4.3), 3–52 8.2 (3.3), 1–15	25.3 (11.2), 9–56 10.9 (3.3), 4–19	29.8 (11.5), 2–67 12.0 (3.4), 1–20

*Number of female/male; Analysis of differences in the gender variable using chi-square test; other analyses using t-test.

mv, missing value.

Regarding clinical and cognitive variables, as expected there were significant differences between groups (*p* < 0.001 for all ANOVAs). For the memory variables (RAVLT immediate and delayed recall and digit span), PwD performed worse than MCI and control participants, and MCI participants performed worse than controls (*p*-values from < 0.001 to < 0.05). For the MMSE, IQCODE, digit to symbol and fluency tasks, PwD performed worse than controls and participants with MCI (*p*-values from < 0.001 to < 0.05), but there were no significant differences between these two groups.

### Differences in activities of daily living

#### Self-report

ANOVA results indicated significant differences between groups for advanced [*F*(2, 211) = 9.77, *p* < 0.001], instrumental [*F*(2, 215) = 27.60, *p* < 0.001] and basic ADL [*F*(2, 216) = 3.70, *p* = 0.026]. Means can be seen in Table 2. *Post hoc* tests indicated that for advanced ADL PwD reported less preserved abilities than controls (*p* < 0.001), but there were no significant differences between participants with MCI and controls (*p* = 0.495) or PwD (*p* = 0.070). Pairwise comparisons of instrumental ADL indicated less preserved abilities for PwD in relation to both MCI and control participants (*p* < 0.001 in both cases), but no significant differences between these two groups (*p* = 0.999). For basic ADL, there were no differences between controls and PwD (*p* = 0.135) or MCI participants (*p* = 0.999), but a significant difference between the latter group and PwD (*p* = 0.036).

**TABLE 2:** Activities of daily living performance and awareness divided by group.

Variable	Dementia (*n* = 104) mean (*SD*), range	MCI (*n* = 45) mean (*SD*), range	control group (*n* = 94) mean (*SD*), range	Differences
*Self-report*				
Advanced ADL^a^ (mv = 29, 0, 0)	3.5 (2.1), 0–8	4.5 (2.7), 1–10	5.1 (2.6), 0–10	PwD = MCI; PwD < CG; MCI = CG
Instrumental ADL^b^ (mv = 25, 0, 0)	15.2 (3.8), 8–21	18.7 (2.1), 13–21	18.5 (3.2), 8–21	PwD < MCI = CG
Basic ADL^c^ (mv = 25, 1, 0)	16.1 (2.5), 6–18	17.2 (1.3), 11–18	16.8 (2.2), 6–18	PwD < MCI; PwD = CG; MCI = CG
*Informant-report*				
Advanced ADL (mv = 1, 3, 7)	2.9 (2.3), 0–8	4.5 (2.5), 1–9	5.1 (2.6), 1–10	PwD < MCI = CG
Instrumental ADL (mv = 0, 0, 6)	11.9 (3.9), 7–21	18.3 (2.4), 13–21	18.2 (3.3), 7–21	PwD < MCI = CG
Basic ADL (mv = 1, 0, 7)	13.8 (4.2), 6–18	17.1 (1.5), 10–18	16.7 (2.3), 6–18	PwD < MCI = CG
*Awareness*				
Advanced ADL (mv = 29, 3, 7)	0.1 (1.6), –5–5	–0.2 (1.6), –6–3	0.0 (1.1), –5–4	PwD = MCI = CG
Instrumental ADL (mv = 25, 0, 6)	2.2 (2.4), –2–9	0.4 (1.1), –1–4	0.2 (1.0), –4–4	PwD = MCI; PwD > CG; MCI = CG
Basic ADL (mv = 25, 0, 7)	0.9 (2.0), –4–8	0.1 (0.5), –1–2	0.1 (0.5), –1–2	PwD > MCI = CG

^a^Reuben’s advanced ADL scale.

^b^Lawton instrumental ADL scale.

^c^The Katz Index of Independence.

mv, missing values.

#### Informant report

ANOVA results indicated significant differences between groups for advanced [*F*(2, 229) = 18.92, *p* < 0.001], instrumental [*F*(2, 234) = 98.76, *p* < 0.001] and basic ADL [*F*(2, 232) = 26.72, *p* < 0.001]. Means can be seen in Table 2. *Post hoc* tests indicated a similar pattern for all types of ADL, with no significant differences between controls and PwMCI (*p* > 0.05), but PwD having less preserved abilities than controls and PwMCI (*p* < 0.001 in all cases).

#### Awareness

ANOVA results indicated significant differences between groups for basic [*F*(2, 206) = 10.57, *p* < 0.001] and instrumental [*F*(2, 209) = 33.68, *p* < 0.001], but not for advanced ADL awareness [*F*(2, 200) = 0.421, *p* = 0.657]. Means can be seen in Table 2. *Post hoc* tests indicated significant differences in awareness of basic ADL abilities between controls and PwD (*p* < 0.001), and between PwMCI and PwD (*p* = 0.002), but not between controls and MCI (*p* = 0.999). Differences in instrumental ADL awareness were significant only between controls and PwD (*p* < 0.001).

### Regression models

There was no evidence of collinearity in the data, with VIF and tolerance values within the recommended range (43). Significant predictors can be seen in Tables 3–5.

**TABLE 3:** Regression models with predictors for ADL self-report scales scores.

	Basic ADL^a^		Instrumental ADL^b^		Advanced ADL^c^	
Variable	β	*P*-value	β	*P*-value	β	*P*-value
Educational level	–0.21	0.008				
Phonemic fluency	0.30	0.003			0.32	<0.001
Age			–0.31	<0.001	–0.24	<0.001
Female gender			0.13	0.034		
Categorical fluency			0.23	0.005		
Immediate recall			0.24	0.029		
Model *p*-value	<0.01		<0.01		<0.01	
*R* ^2^	0.13		0.39		0.28	
Adjusted *R*^2^	0.10		0.37		0.27	

ADL, Activities of Daily Living; Basic ADL, The Katz Index of Independence; Instrumental ADL, Lawton. instrumental ADL scale; Advanced ADL, Reuben’s advanced ADL scale.

^a^ The Katz Index of Independence.

^b^ Lawton instrumental ADL scale.

^c^ Reuben’s advanced ADL scale.

**TABLE 4:** Regression models with predictors for ADL informant-report scales scores.

	Basic ADL^a^		Instrumental ADL^b^		Advanced ADL^c^	
Variable	β	*P*-value	β	*P*-value	β	*P*-value
Educational level	–0.25	0.002	–0.15	0.011		
Phonemic fluency	0.26	0.007			0.32	<0.001
Diagnostic group			–0.18	0.016		
MMSE score			0.16	0.023		
Categorical fluency			0.18	0.011		
Immediate recall			0.17	0.025		
Female gender			0.15	0.009	0.14	0.036
Age			–0.25	<0.001	–0.25	<0.001
Model *p*-value	<0.001		<0.001		<0.001	
*R* ^2^	0.15		0.54		0.30	
Adjusted *R*^2^	0.12		0.52		0.28	

ADL, Activities of Daily Living; Basic ADL, The Katz Index of Independence; Instrumental ADL, Lawton instrumental ADL scale; Advanced ADL, Reuben’s advanced ADL scale; MMSE, Mini-Mental State Examination.

^*a*^ The Katz Index of Independence.

^*b*^ Lawton instrumental ADL scale.

^*c*^ Reuben’s advanced ADL scale.

**TABLE 5:** Regression models with predictors for ADL awareness.

	Basic ADL^a^		Instrumental ADL^b^	
Variable	β	*P*-value	β	*P*-value
Delayed recall	–0.20	0.018		
Educational level			0.16	0.036
Diagnostic group			0.31	0.001
MMSE score			–0.20	0.035
Model *p*-value	<0.01		<0.01	
*R* ^2^	0.09		0.27	
Adjusted *R*^2^	0.07		0.24	

ADL, Activities of Daily Living; Basic ADL, The Katz Index of Independence; Instrumental ADL, Lawton instrumental ADL scale; MMSE, Mini-Mental State Examination.

^*a*^The Katz Index of Independence.

^*b*^Lawton instrumental ADL scale.

#### Self-report

All regression models significantly predicted advanced, instrumental and basic ADLs (*p* < 0.01 in all models). For advanced ADL, the model with the best trade-off between AIC score, explained variance (*R*^2^ = 0.28) and highest cross-validity (adjusted *R*^2^ = 0.27) included phonemic fluency (standardized β = 0.32, *p* < 0.001), age (standardized β = –0.24, *p* < 0.001), and categorical fluency, but the latter variable did not give a significant contribution to the model (*p* = 0.181).

For instrumental ADL, the model with the best (lowest) AIC score, highest explained variance (*R*^2^ = 0.39) and highest cross-validity (adjusted *R*^2^ = 0.37) included female gender (standardized β = 0.13, *p* = 0.034), age (standardized β = –0.31, *p* < 0.001), categorical fluency (standardized β = 0.23, *p* = 0.005), immediate recall (standardized β = 0.24, *p* = 0.029), and variables which did not give a significant contribution to the model, such as phonemic fluency (*p* = 0.194), delayed recall (*p* = 0.371) and educational level (*p* = 0.182).

For basic ADL, the model with the best (lowest) AIC score, highest explained variance (*R*^2^ = 0.13) and highest cross-validity (adjusted *R*^2^ = 0.10) included educational level (standardized β = –0.21, *p* = 0.008), phonemic fluency (standardized β = 0.30, *p* = 0.003) and variables which did not give a significant contribution, such as age (*p* = 0.051), diagnosis (*p* = 0.387), categorical fluency (*p* = 0.563) and delayed recall (*p* = 0.064).

#### Informant-report

All regression models significantly predicted advanced, instrumental and basic ADLs (*p* ≤ 0.001 in all models). For advanced ADL, the model with the best (lowest) AIC score, highest explained variance (*R*^2^ = 0.30) and highest cross-validity (adjusted *R*^2^ = 0.28) included phonemic fluency (standardized β = 0.32, *p* < 0.001), female gender (standardized β = 0.14, *p* = 0.036), age (standardized β = –0.25, *p* < 0.001), and variables which did not give a significant contribution to the model, such as categorical fluency (*p* = 0.297) and educational level (*p* = 0.514).

For instrumental ADL, the model with the best trade-off between AIC score, highest explained variance (*R*^2^ = 0.54) and highest cross-validity (adjusted *R*^2^ = 0.52) included all variables, except delayed recall, with significant contributions of diagnosis (standardized β = –0.18, *p* = 0.016), female gender (standardized β = 0.15, *p* = 0.009), age (standardized β = –0.25, *p* < 0.001), educational level (standardized β = –0.15, *p* = 0.011), categorical fluency (standardized β = 0.18, *p* = 0.011), MMSE (standardized β = 0.16, *p* = 0.023), immediate recall (standardized β = 0.17, *p* = 0.025), but not of phonemic fluency (*p* = 0.056) or digit span (*p* = 0.199).

For basic ADL, the model with the best trade-off between AIC score, highest explained variance (*R*^2^ = 0.15) and highest cross-validity (adjusted *R*^2^ = 0.12) included educational level (standardized β = –0.25, *p* = 0.002), phonemic fluency (standardized β = 0.26, *p* = 0.007) and variables which did not give a significant contribution, such as age (*p* = 0.060), categorical fluency (*p* = 0.391), MMSE (*p* = 0.360) and delayed recall (*p* = 0.708).

#### Awareness

The regression models significantly predicted instrumental and basic ADL awareness (*p* ≤ 0.01 in all models), but not advanced ADL awareness. For instrumental ADL awareness, the model with the best trade-off between AIC score, highest explained variance (*R*^2^ = 0.27) and highest cross-validity (adjusted *R*^2^ = 0.24) included diagnosis (standardized β = 0.31, *p* = 0.001), educational level (standardized β = 0.16, *p* = 0.036), MMSE (standardized β = –0.20, *p* = 0.035) and variables which did not give a significant contribution, such as digit span (*p* = 0.120), phonemic fluency (*p* = 0.306), and delayed recall (*p* = 0.082).

For basic ADL, the model with the best trade-off between AIC score, highest explained variance (*R*^2^ = 0.09) and highest cross-validity (adjusted *R*^2^ = 0.07) included only delayed recall as a significant predictor (standardized β = –0.20, *p* = 0.018), and variables which did not give a significant contribution, such as educational level (*p* = 0.095), categorical fluency (*p* = 0.318), and MMSE (*p* = 0.276).

## Discussion

Results have shown more impairments in all ADL levels for PwD. Considering self-reported ability, PwD were more impaired than controls in advanced and instrumental activities and more impaired than PwMCI in instrumental and basic activities. In the informant-reported measures, PwD were more impaired than both groups in all types of ADL. No differences were seen between controls and PwMCI regardless of type of measure. Age, gender, education and fluency were the most consistent predictors for ADL performance, across measurement types and level of complexity. Regarding awareness, for advanced ADL there were no differences between groups and no significant regression model. By contrast, PwD showed decreased awareness relative to controls for instrumental and basic, and relative to MCI for basic ADL. Memory was the only predictor for basic ADL awareness, while diagnosis, general cognitive status and educational level were significant predictors for instrumental ADL awareness.

Significant differences found between controls and PwD are in line with standard findings (22). ADL performance gradually decreases with progression of dementia, eventually affecting even basic activities (1, 3). Although all levels of ADL performance were significantly different concerning informant-report, it is important to highlight that the differences in self-reported measures between PwD and controls did not include basic activities. A possible explanation is related to unawareness within the PwD group, also found in other studies [e.g., (44)]. Because basic activities are the last to be impaired in the course of dementia, cognitive deficits may have already affected the subjects’ awareness by then (4). In this case, informants perceive the deficit, but not PwD, who overestimate their performance, attenuating group differences. This notion is supported by PwD showing poorer awareness of ability relative to controls for both instrumental and basic ADL in the current study.

Regarding advanced ADL, in the self-report measures PwD are impaired only in relation to controls, whilst for informant-report PwD have lower ability in relation to both controls and PwMCI. Again, this suggests that informant-report may be more reliable to ascertain functional change. Direct comparison of awareness of advanced ADL between groups did not show significant differences, but it is possible this was caused by generally low scoring in the variable (i.e., a floor effect).

Lack of significant differences in ADL between control participants and PwMCI is consistent with the definition of this condition, with MCI being characterized by cognitive impairment in the absence of functional deficits (2). Nevertheless, this has been recently questioned in the literature (12, 17). It is possible that the measures used may not be sensitive enough to detect subtle functional changes (45), with impairments at this level affecting the processes more than their results, making difficulties less noticeable (15, 16). Results indicate that PwMCI show good awareness of their functional abilities, with consistent scores regardless of type of report, something that has been reported previously (46). PwMCI also did not show significant differences in relation to controls in any of the awareness variables, with mean values very close to zero, suggesting accurate assessment of ability.

Cognitive and demographic predictors of functional ability were generally consistent across measurement type and ADL level. Higher phonemic fluency and lower age predicted better advanced ADL for both measurement types; in the informant variable, women showed better functional capacity. Older age may lead to decreases in advanced ADL since it is the greatest risk factor for cognitive decline (13). By its turn, cognitive decline causes loss of functional capacity (1), which starts with advanced activities (11).

The presence of phonemic fluency in the models may suggest cognitive processes that are at some level linked to advanced ADL performance. This statement, however, should be considered with three caveats. First, fluency tests are influenced by a variety of different cognitive processes (47, 48), and also, potentially, demographic variables [e.g., age and educational level; (45), lacking in specificity]. Second, the absence of other cognitive variable suggests that other processes, not cognitive in nature, may be involved [e.g., mood disorder (4)]. Third, in a similar sense, phonemic fluency is a test highly sensitive to conditions that affect the frontal lobes (49) and processing speed (47), which can be linked to mood changes, for instance (50, 51).

In the informant report, gender was also a predictor, with women showing better functional ability. Although biological hypotheses could be made, this finding may be more readily explained by differences in perception due to psychosocial and cultural roles. Men usually engage more in work-life (52) and less in hobbies, groups and social activities in life, continuing with this pattern in older age (53). Diminished activities can be seen as normal by men themselves, being reported differently by caregivers.

Instrumental activities abilities were, in both models, also predicted by age and gender, as well as by categorical fluency, immediate recall and education. Diagnosis and MMSE were present in the informant-report model as well. Age and gender are present in this model probably for the same reasons they were present as predictors for advanced activities. The presence of gender for both informant and self-report may be explained by the fact that men are commonly and consciously less involved in housework than women (52), with instrumental activities measures focusing precisely on this type of activity.

Instrumental ADL models included more cognitive variables than for advanced and basic ADL. Both self- and informant-report models included categorical fluency and immediate recall. This is consistent with previous findings that relate instrumental abilities primarily to executive functioning and memory (3, 45). Categorical fluency is also related to other cognitive functions (47, 48) and demographic variables such as age and education (54). Regarding this and that informant-report model includes also the variables MMSE and diagnosis, a possible conclusion is that instrumental activities performance depends highly on global cognitive status, as has already been indicated in the literature (7, 14, 15, 45, 55). It is worth highlighting the fact that diagnosis was a predictor only for instrumental ADL, which suggests the diagnostic value of this variable when identifying PwD from those with MCI and healthy older adults. This also suggests that although dementia diagnosis contributes to disability across activities type, other factors, such as age, are stronger predictors of functional capacity.

Education is a variable that may often be undetected as a predictor of functional capacity because most studies about the theme have been conducted in developed countries, characterized by higher educational homogeneity. In this study, the variable appeared in instrumental and basic ADL models, both in self- and informant- report. Higher education is one of the factors known to provide higher cognitive reserve (56), a protection against cognitive impairment (56, 57) and consequently against its consequences such as functional loss (1).

In addition to education, both basic ADL prediction models included also phonemic fluency. First, it is important to highlight that phonemic fluency is associated with education (54). It has also been shown to be associated with diverse cognitive processes, such as language, executive functions and processing speed (47, 48). Current findings highlight the fact that cognitive impairment can be linked to poorer basic ADL performance (1), despite lower complexity to perform these activities.

Regarding awareness, general cognitive level was a significant predictor of instrumental ADL awareness, and memory was the only predictor of awareness of basic ADL, although explained variance was low for the latter. Altogether these findings suggest that awareness in can be considered a neurocognitive ability, as proposed by theoretical models (58). Additionally, awareness of instrumental ADL was also predicted by diagnosis and educational level. This reinforces the notion of lack of awareness as a marker of dementia (19), also suggesting that wider social awareness, including access to educational resources and general knowledge about the condition, may impact on self-perception of ability (59). In agreement with the current study, associations between awareness in dementia and educational level have been reported before [e.g., (56)]. There were no significant models for awareness of advanced ADL, which can be explained either by variables not included in the model, such as mood and personality playing a role in perception of advanced ADL, or by lack of variance for this variable in the current study.

One limitation of the current study refers to the measurement of the main variables explored. The neuropsychological test battery could have been more diverse, exploring also cognitive abilities such as inhibitory control and planning. Nevertheless, a briefer battery was chosen considering application time and the populational approach used in the current study. Questionnaire measurements of ADL also may be criticized for reduced ecological validity, and, ideally, observational measures should be applied. Nevertheless, in the current setting, such measures were not possible, so future community-based work should consider the applicability of ADL outcomes with increased ecological validity. Another limitation is the lack of information regarding specific diagnosis for all participants within the dementia group. Different types of dementia could have led to distinct profiles in terms of ADL and awareness impairment. Nevertheless, where information is available, most participants had Alzheimer’s disease, the most common form of dementia in older adults. Future studies could explore ADL performance and awareness, as well as their predictors, in different types of dementia.

## Conclusion

In conclusion, the results imply lack of awareness of ADL ability, as well as poorer performance, in PwD, with a subtle decrease in performance in PwMCI in advanced activities. As informant-report consistently showed differences between PwD and both other diagnostic groups in all ADL levels, using informant measures may be crucial for clinical assessment of functional capacity. Using ADL screening may be especially important to LMIC countries, where more expensive methods are not available (60). Regarding cognition, testing fluency can also have an important role as it appeared as a predictor of all ADL types of performance. Even though cognition may play a smaller role in advanced ADL abilities, instrumental abilities are the most consistently affected within each diagnostic group. This finding suggests that the latter level may be the one most directly and purely affected by global cognitive impairment, which is reinforced by the inclusion of diagnosis as a significant predictor. Finally, it is worth highlighting the finding about the relationship between educational level and ADL, considering that this factor may be often overlooked in more developed regions. This suggest that educational achievement, as well as improving cognitive reserve, may, potentially, have a direct impact in functional capacity, warranting further studies in developing countries.

## Data availability statement

The raw data supporting the conclusions of this article will be made available by the authors, upon reasonable request.

## Ethics statement

Stage I of the FIBRA study was approved by the Pedro Ernesto University Hospital Ethics Committee (1850-CEP HUPE/2007), with subsequent approval for stage II (0163.2008-COEP UERJ 027/2008). All participants and their caregivers provided informed consent.

## Author contributions

RL was responsible for the conception and design of the study. CF and PR were responsible for data acquisition and the database organization. LH was responsible for the writing. DM and LH were responsible for data analysis. DM, LH, HF, MS, and RL contributed to the interpretation. All authors contributed to the manuscript revision, read, and approved the submitted version.

## Conflict of interest

The authors declare that the research was conducted in the absence of any commercial or financial relationships that could be construed as a potential conflict of interest.

## Publisher’s note

All claims expressed in this article are solely those of the authors and do not necessarily represent those of their affiliated organizations, or those of the publisher, the editors and the reviewers. Any product that may be evaluated in this article, or claim that may be made by its manufacturer, is not guaranteed or endorsed by the publisher.
